# Art of Disaster Preparedness in European Union: a Survey on the Health Systems

**DOI:** 10.1371/currents.dis.56cf1c5c1b0deae1595a48e294685d2f

**Published:** 2014-12-17

**Authors:** Ahmadreza Djalali, Francesco Della Corte, Marco Foletti, Luca Ragazzoni, Alba Ripoll Gallardo, Olivera Lupescu, Chris Arculeo, Götz von Arnim, Tom Friedl, Michael Ashkenazi, Philipp Fischer, Boris Hreckovski, Amir Khorram-Manesh, Radko Komadina, Konstanze Lechner, Cristina Patru, Frederick M. Burkle, Pier Luigi Ingrassia

**Affiliations:** CRIMEDIM - Research Center in Emergency and Disaster Medicine and Computer Science applied to Medical Practice; Università del Piemonte Orientale, Novara, Italy; CRIMEDIM - Research Center in Emergency and Disaster Medicine and Computer Science applied to Medical Practice; Università del Piemonte Orientale, Novara, Italy; CRIMEDIM - Research Center in Emergency and Disaster Medicine and Computer Science applied to Medical Practice; Università del Piemonte Orientale, Novara, Italy; CRIMEDIM - Research Center in Emergency and Disaster Medicine and Computer Science applied to Medical Practice; Università del Piemonte Orientale, Novara, Italy; CRIMEDIM - Research Center in Emergency and Disaster Medicine and Computer Science applied to Medical Practice; Università del Piemonte Orientale, Novara, Italy; URGENTA, Clinical Emergency Hospital, Bucharest, Romania; Hanover Associates, Teddington, London, United Kingdom; NHCS, National Health Career School of Management, Hennigsdorf/Berlin, Germany; NHCS, National Health Career School of Management, Hennigsdorf/Berlin, Germany; Bonn International Center for Conversion, Bonn, Germany; University Clinic Bonn, Department of Orthopedics and Trauma Surgery, Bonn, Germany; CROUMSA, Croatian Urgent Medicine and Surgery Association, Slav. Brod, Croatia; Prehospital and Disaster Medicine Centre, Sahlgrenska Academy, Gothenburg, Sweden; SBC, General &Teaching Hospital Celje, Medical Faculty Ljubljana, Slovenia; German Aerospace Center (DLR), Oberpfaffenhofen, Germany; Clinical Emergency Hospital Bucharest, Romania; Harvard Humanitarian Initiative, Harvard School of Public Health, Harvard University, Cambridge, Massachusetts, USA; CRIMEDIM - Research Center in Emergency and Disaster Medicine and Computer Science applied to Medical Practice; Università del Piemonte Orientale, Novara, Italy

## Abstract

Introduction: Naturally occurring and man-made disasters have been increasing in the world, including Europe, over the past several decades. Health systems are a key part of any community disaster management system. The success of preparedness and prevention depends on the success of activities such as disaster planning, organization and training. The aim of this study is to evaluate health system preparedness for disasters in the 27 European Union member countries. Method: A cross-sectional analysis study was completed between June-September 2012. The checklist used for this survey was a modified from the World Health Organization toolkit for assessing health-system capacity for crisis management. Three specialists from each of the 27 European Union countries were included in the survey. Responses to each survey question were scored and the range of preparedness level was defined as 0-100%, categorized in three levels as follows: Acceptable; Transitional; or Insufficient. Results: Response rate was 79.1%. The average level of disaster management preparedness in the health systems of 27 European Union member states was 68% (Acceptable). The highest level of preparedness was seen in the United Kingdom, Luxemburg, and Lithuania. Considering the elements of disaster management system, the highest level of preparedness score was at health information elements (86%), and the lowest level was for hospitals, and educational elements (54%). Conclusion: This survey study suggests that preparedness level of European Union countries in 2012 is at an acceptable level but could be improved. Elements such as hospitals and education and training suffer from insufficient levels of preparedness. The European Union health systems need a collective strategic plan, as well as enough resources, to establish a comprehensive and standardized disaster management strategy plan. A competency based training curriculum for managers and first responders is basic to accomplishing this goal. Keywords: Disaster medicine; Disaster preparedness; Disaster epidemiology; Health systems; European Union

## Introduction

Over the past several decades, naturally occurring and man-made disasters have increased in frequency and number, worldwide. In Europe, during the period 1980-2008, around 122,000 people were killed and 33 million negatively affected because of natural disasters.^1^ Countries such as France, Italy and Spain have been greatly impacted by disasters, while Sweden, Norway and Denmark have not suffered from major events.^1^^,^ 2

The nation-states’ health system is a key component of disaster management organization, and preparedness status of the health system is essential for an effective response to disasters.^3^^,^ 4 Where national and local health systems are not well prepared to deal with disasters, vulnerability and resilience at the community level becomes more pronounced.^5^

Preparedness is defined as “the knowledge and capacities developed by governments, professional response and recovery organizations, communities and individuals to effectively anticipate, respond to, and recover from, the impacts of likely, imminent or current hazard events or conditions”.^6^ Preparedness is achieved through a set of activities and foundations, such as planning, organization and training.^7^

Disaster Training Curriculum (DITAC) is a research project funded by the European Commission under the 7th Framework Program. The main aim of this project is to develop a holistic and standardized training curriculum for first responders and strategic/tactical crisis managers in the 27 European Union (EU) member states to enhance the level of preparedness in these countries.

The development of a standardized competency-based training curriculum requires a clear and comprehensive understanding of the condition of the disaster management system in the target communities. The first phase of the DITAC project and of this survey study is to evaluate the current state of preparedness and disaster management in all 27 EU countries.

## Methods

This study is an observational, cross-sectional study. The survey was conducted between June and September 2012. All 27 EU countries were included in this study.

A standardized online survey instrument was developed and hosted on SurveyMonkey (SurveyMonkey LLC, Palo Alto, California USA). Survey design utilized a similar model from the toolkit for assessing health-system capacity for crisis management of the World Health Organization / European office.^8^ On the basis of experts’ consensus, 88 questions of disaster management relevance of the toolkit were selected and modified, to ensure optimal feasibility, for this survey. The questions were grouped in the following elements:

- Leadership and governance

- Logistics and operational support functions in emergencies

- Medical products and technology

- Health information

- Sub-national/Regional plans for crisis/MCIs

- Management of pre-hospital medical operations

- Hospital emergency-preparedness program

- Education and training

The survey questionnaire was delivered online to 3 professionals from each of the 27 EU countries identified as having inclusion criteria of 5 years of professional experience and tertiary level of education in health system management. These selectively included the president of the counties’ emergency medicine society, a health system stakeholder, and an expert in emergency management. All professionals were involved in various disciplines of health-system disaster preparedness and management, such as Emergency Medical Services, hospitals, and governmental offices for disaster management.

Answer to the questions was scored as 0 for No, 1 for partially complete and 2 as totally completed, however some questions were not scored because they were complementary questions to provide more details on the given element. The range of preparedness level was defined as 0-100%, categorized in three levels as: A (Acceptable), B (Transitional), C (Insufficient). The required action for each level was defined in Table 1.

**Table 1- Classification of health system preparedness, based on the total score, and the required action:** 

The score of preparedness	The level of preparedness	Required action
66-100%	Level A: Acceptable	It is likely that the disaster management system will effectively function in a disaster. It is recommended, however, to continue with measures to improve the preparedness level.
36-65%	Level B: Transitional	The preparedness of disaster management system will not be able to operate effectively during and after a disaster. Interventional measures are needed.
0-35%	Level C: Insufficient	Current preparedness of the disaster management system is impaired and unreliable during and after a disaster. Urgent intervention is needed.

Informed consent was obtained and all participants were informed they could refuse to participate or withdraw from the study at any time. Also, name, personal information and affiliation data of the participants were kept confidential.

## Results

The questionnaire was sent to 81 people in 27 EU countries. Response rate was 79.1%, which represented at least one person per country. Twenty four percent of the respondents were female.

In this study, the self-assessed average level of disaster management preparedness in the health systems of the 27 EU member states was 68%, classified as level A (Acceptable). Nevertheless, the level of preparedness was diverse among the EU countries (Figure 1). The highest level of preparedness was seen in the United Kingdom, Luxemburg, and Lithuania, as 84%, 84% and 83%, respectively. Portugal, Malta and Ireland reported lowest scores, as 42%, 44%, and 51%, respectively.

**The level of preparedness, in respect of disaster management, in 27 European Union countries:**
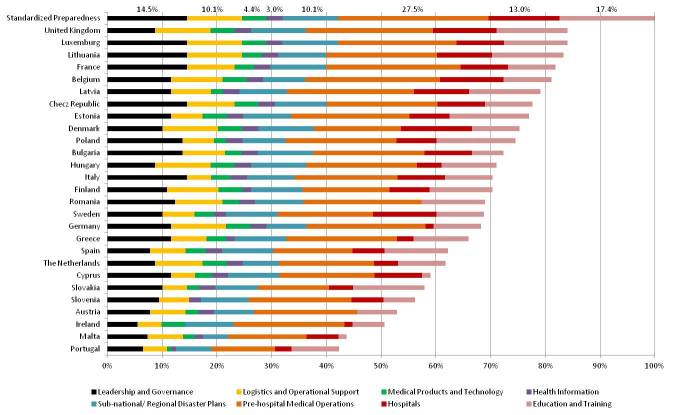

Two-thirds of EU countries were at acceptable level of disaster preparedness, and one-third were at transitional level. No country was categorized as having an insufficient level of preparedness in disaster management.

In EU country respondents, with regard to different elements of the disaster management system, the highest level of preparedness was seen in the health information element (86%), and the lowest level of preparedness was seen at both hospitals and education and training elements (54%) (Figure 2).

**Preparedness level of different element of disaster management system in whole European Union:**
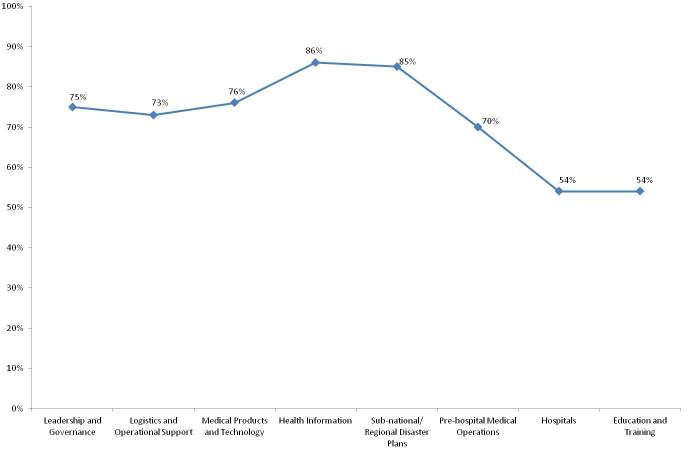

Preparedness of EU respondents was categorized as “Acceptable”: a) leadership and governance; b) logistics and operational support;c) medical products and technology; d) health information; e) sub-national/ regional disaster plans; and f) pre-hospital medical operations.

On the other hand, preparedness was considered “Transitional” for elements: g) hospitals; and h) education and training, in whole EU.

No element was at the “Insufficient” preparedness level.

Existence of each of 88 preparedness items, categorized in 8 main elements, either completely or partially in EU health systems is summarized as Appendix 1.

## Discussion

At least 73% of EU countries have recently experienced an emergency or disaster.^9^ This demonstrates the importance of health emergency preparedness, prevention and response programs in Europe. Preparedness actions aim to build resiliency and capacity needed to efficiently and effectively manage all types of emergencies.^6^

This study, based on survey measures, shows evidence that the level of disaster management preparedness in the EU is considered “acceptable”, overall, by a slim level compared to the lower “transitional” level. Although similar EU-wide evaluation has not been reported, current results are supported by findings of a worldwide survey on health system preparedness by the WHO in which most of evaluated criteria were categorized “acceptable”, within 40%-80%.^9^ Also, a study on decontamination capability of medical systems showed a “weak” preparedness situation in EU member states.^10^

One can conclude that Europe overall lacks in the safety elements surveyed with regard to disaster occurrence. Around 1,200 naturally occurring disasters have happened in Europe within the past 3 decades which have affected millions of inhabitants.^1^ In case of a disaster, the most important goal for a community is the health and well-being of its people. Current borderline status of disaster preparedness in the EU health systems, especially those with transitional level of preparedness, needs intensive attention to reach at more reliable level.

The results of this survey supports that the health systems in EU are considered well prepared for disasters, by a cross section of professional disaster managers within their own countries, with respect to leadership and governance, logistics and operational support, medical products and technology, health information, pre-hospital medical operations, and planning at sub-national/ regional level.

On the basis of the authors’ knowledge, there is no similar published report on disaster preparedness of the health systems in all EU countries that either support or deny the survey study conclusions. However, it is necessary for EU member states to sustain the activities and plans to enhance health system readiness, even those elements that appear to be well-prepared. These efforts should be completed at national, regional and local levels.

On the basis of this survey, hospitals in EU countries are not well prepared to face disasters. This result is consistent with other studies, which show lack of preparedness in some hospitals of EU member states.^11^^-^ 14 Hospitals are cornerstone of health systems, both during daily life and in disasters. In fact, hospitals are essential facilities for the maintenance of vital societal functions, specifically health and well-being of the population. Therefore the disruption or destruction of hospitals will have a significant impact in the member state outcomes as a result of the failure to maintain those vital functions.

It is critical for all EU communities to have well prepared hospitals to provide reliable medical services for victims and affected population. The use of standardized guidelines, such as WHO toolkits, may help the hospitals to reach an “acceptable” level of disaster preparedness. In addition, allocation of financial resources is crucial to the establishment and functioning of disaster preparedness and response programs in the health systems, including hospitals.^8^ It is recommended that the EU member states give a higher priority to financial support for hospital disaster preparedness programs.

This study suggests that there is a lack of education and training in disaster preparedness in the health systems of EU countries. In addition, the curricula and training materials are still not well harmonized across different stakeholders.Previous studies support this finding.^4^^,^ 15

Recognizing that education and training are essential elements of capacity building and a sense of resiliency in disaster management, the development of multidisciplinary core competencies, on the basis of international guidelines and standards, is a necessity for the EU health system to adopt in order to enhance the capacity for disaster preparedness.^16^^-^ 19

**Limitations**: One limitation of the current study was the small sample size and sampling method. The position, experience and background of respondents are general in nature and self-determined. This factor may affect the validity and reliability of the collected information. However, this is a comprehensive study that included all EU member states. The results can be a useful baseline for future comparisons and be helpful for identifying gaps in disaster preparedness planning.

A second limitation is that the study data were gathered, analyzed and categorized on the basis of a survey questionnaire which had not been validated to ensure if it reflects measurable reality with respect to disaster management preparedness. However, there was consensus of the experts on the questionnaire items, and the study benefitted from the standardized toolkit of WHO that served as a model for this survey.

Finally, to ask participants to report the preparedness elements of their own country could result is some level of bias, either overestimation or underestimation of the preparedness level. However, it was not possible and acceptable to ask an expert to evaluate another country, in respect of disaster preparedness, through this survey. Also, all participants were experts in the health disaster field and well familiar with the status of their country. Therefore, the results should be accepted as realistic and valid.

## Conclusion

This study showed that the preparedness level of EU countries is barely considered at an “acceptable” level. This should alert the EU country disaster and health system professionals to do more. Although some functions such as health information and pre-hospital system are at high level of preparedness, hospitals overall suffer from an insufficient level of preparedness. A lack of competency-based training and education is the main gap in health disaster preparedness in the EU.

The EU health system, in our considered opinion, requires a comprehensive strategic policy, as well as sufficient resources, to establish a comprehensive and standardized disaster management plan. A competency-based training curriculum for managers and first responders is a necessary first step basic action to reach that goal.

## Competing Interests

The authors have declared that no competing interests exist.

## Appendix 1

### Existence of disaster preparedness elements in the health system of 27 EU member states, described as 88 items categorized in 8 groups

**Table:**

Preparedness element	Evaluated items	Condition in EU*
**Leadership and governance**	Legislation reference to crisis/disaster management	100%
	Legislation follows all-hazards approach	93%
	Legislation includes activation, coordination and incident command mechanisms	96%
	Coordination and incident command system is regularly tested through exercises, drills and simulations	60%
	A structure for interagency coordination for disaster/MCI management (either in national level or/and in regional level)	96%
	How is the structure	Explanation
	National operational crisis centre in case of a disaster (EOC)	§ Ministry of Interior: 56% § Other organizations: 44%
	Medical and public health representation in the EOC	96%
	The responsible organization for coordination of response in case of a MCI at regional level	§ Local authority: 50% § Fire Bridge: 19% § Other organizations: 31%
	The legislation recognizes binding international (bilateral/multilateral) agreements and conventions	85%
	Regulations relating to the entry of foreign health workers to provide emergency relief services	50%
	Multi-sectoral financing procedures available for the request, acceptance and utilization of international financial assistance	68%
	Multi-sectoral financing mechanisms include contingency funding for response and recovery at the national and regional levels	70%
**Logistics and operational support functions in emergencies**	Guidelines and procedures for establishing standardized telecommunications systems across all sectors	93%
	Protocols for the use of temporary means of telecommunication	85%
	The staff are trained to use the telecommunications equipment in emergencies	96%
	Guidelines and procedures exist for the use and management of logistics systems in emergency situations	96%
	The staff are trained to use the logistics systems in emergencies	92%
	Available resources to ensure logistics support in emergencies	96%
	Agreements in place with partners and/or private companies for the provision of logistics services to ensure continuity of essential functions	81%
**Medical products and technology**	Essential medical supplies and equipment for emergency operations determined on the basis of risk assessments and analyses	93%
	The essential medical supplies and equipment are readily available in sufficient quantities/stockpiles	89%
	The supplies and equipment are periodically checked and tested, in accordance with established guidelines	93%
**Health information**	The responsibilities and authority related to the information system are defined	88%
	Early-warning capacity is in place to enable recognition of and reporting on any event of potential public health concern within 24 hours	96%
**Sub-national/ Regional plans for crisis/ MCIs**	Sub-national/regional emergency response plans for health system, based on national policy	96%
	The health plans are compatible with the community multi-sectoral emergency plan	96%
	The plans define mechanisms for activation, coordination, command and control	96%
	The plans are regularly tested, validated, exercised and maintained	96%
	Mechanisms exist for a rapid mobilization of additional resources (personnel, equipment and materials) among sub-national levels	96%
	Mechanisms of networking exist	96%
	Procedures and the required capacity (ventilators, incubators, etc.) exist for providing life support and critical care during patient transport outside the affected area	100%
**Management of prehospital medical operations**	The EMS is under authority of	§ Public government: 78% § Mixed jurisdiction: 22%
	The EMS manage the health assistance in case of a MCI and/or disaster	89%
	If not, there is another office to manage a MCI and/or disaster	11%
	By whom is it activated	§ Different focal points
	The EMS has a budget line for crisis preparedness	69%
	The EMS has a reserve budget for prompt mobilization to use in case of disasters	56%
	There is a specific agency for health aspects related only to non-terrorism CBRNE events	31%
	The above agency is at	§ National level: 27% § Regional level: 4%
	There is a specific agency for health aspects related only to terroristic event	11%
	The above agency is at	§ National level: 11%
	There is a standardized system in place for managing medical activities at the scene	93%
	There is a standardized triage system in place at prehospital level	81%
	The mentioned triage system is	§ The most common method is START
	The search and rescue operations include a medical component	88%
	Specific arrangements are in place, at prehospital system, to manage contagious and contaminated casualties	85%
	The role of EMS system in identifying and reporting unusual public health events clearly is defined	67%
	The EMS is included in coordination meetings, joint exercises, drills and training exercises	100%
	There is a telephone number for medical emergencies	100%
	It works either for daily emergencies and Crisis Dispatch	100%
	It is nationwide?	100%
	The EMS is obliged by law to have crisis preparedness plan	89%
	The incident commander in case of a MCI	§ A specific chief commander: 58% § An operations management group: 42%
	The specific commander is	§ Fire Brigade: 58% § Police: 16% § EMS: 10% § Other: 16%
	The incident commander in case of a disaster	§ A specific chief commander: 33% § An operations management group: 67%
	The specific commander is	§ Fire Brigade: 63% § Police: 19% § EMS: 6% § Other: 12%
	A difference in deployment between different incidents	54%
	The mentioned difference is because of	§ Type of incident, e.g. CBRN, Train accident, Terrorism, etc
	The interagency communication system is defined as well working	81%
	If not, because	§ No on-site inter-connectivity between police, fire brigade and EMS; different system of communication using by response organizations; different radio frequencies, etc.
	A national system adopted for triage	77%
	If yes, it is working at prehospital setting	37%
**Hospital emergency preparedness program**	A formal hospital emergency preparedness program	82%
	Specific fund is allocated to the emergency preparedness program	38%
	The program fully incorporate the concept of safe hospital	50%
	Specific plan is dedicated to chemical incidents	70%
	The hospitals have decontamination capability stated by law	19%
	The hospitals have planning committees for emergency response and recovery by law	62%
	A plan for emergency response and recovery is a requirement for hospital accreditation	50%
	The hospital plan for emergency response and recovery is validated and accredited in accordance with national criteria	62%
	The hospital plan is reviewed, exercised, revised and updated regularly	44%
**Education and training**	Emergency medicine is a distinct medical specialty	63%
	If yes, it is established within	§ 5 years ago § More than 5 years ago
	A disaster medicine curriculum is formally included in residency programs	54%
	If yes, the residency programs are	§ Emergency medicine § Anaesthesia and intensive care
	A disaster medicine curriculum is formally included in undergraduates programs	44%
	The disaster medicine curriculum for undergraduate students is mainly based on a University proposal	75%
	A post-graduate training in disaster medicine for doctors working in EMS	70%
	If yes, it takes ... months	§ <6 months: 53% § 6-12 months: 29% § >12 months: 18%
	Also, is it based on a specific course or curriculum	38%
	It is left to the initiative of the single region/institution	57%
	There is specific center for education and training in Disaster medicine	81%
	Post-graduate training in disaster medicine for nurses working in EMS	74%
	Opportunities provided for emergency-management training	82%
	The curricula and training materials are harmonized across stakeholders	31%
	The training programs for stakeholders include exercises and drills	70%
	Sufficient resources have been allocated for training programmes	15%

***** **The item is in the place, either completely or partially**
